# The Effects of Polyaniline Nanofibers and Graphene Flakes on the Electrical Properties and Mechanical Properties of ABS-like Resin Composites Obtained by DLP 3D Printing

**DOI:** 10.3390/polym15143079

**Published:** 2023-07-18

**Authors:** Somi Jang, Sunghun Cho

**Affiliations:** 1 School of Chemical Engineering, Yeungnam University, Gyeongsan 38541, Republic of Korea; 2 Department of Materials Science and Engineering, Research Institute of Advanced Materials, Seoul National University, Seoul 08826, Republic of Korea

**Keywords:** 3D printing, digital light processing, ABS-like resin, polyaniline, graphene

## Abstract

Three-dimensional printing is regarded as a future-oriented additive manufacturing technology that is making significant contributions to the field of polymer processing. Among the 3D printing methods, the DLP (digital light processing) technique has attracted great interest because it requires a short printing time and enables high-quality printing through selective light curing of polymeric materials. In this study, we report a fabrication method for ABS-like resin composites containing polyaniline (PANI) nanofibers and graphene flakes suitable for DLP 3D printing. As-prepared ABS-like resin composite inks employing PANI nanofibers and graphene flakes as co-fillers were successfully printed, obtaining highly conductive and mechanically robust products with the desired shapes and different sizes through DLP 3D printing. The sheet resistance of the 3D-printed composites was reduced from 2.50 × 10^15^ ohm/sq (sheet resistance of pristine ABS-like resin) to 1.61 × 10^6^ ohm/sq by adding 3.0 wt.% of PANI nanofibers and 1.5 wt.% of graphene flakes. Furthermore, the AP3.0G1.5 sample (the 3D-printed composite containing 3.0 wt.% of PANI nanofibers and 1.5 wt.% of graphene flakes) exhibited 2.63 times (22.23 MPa) higher tensile strength, 1.47 times (553.8 MPa) higher Young’s modulus, and 5.07 times (25.83%) higher elongation at break values compared to the pristine ABS-like resin with a tensile strength of 8.46 MPa, a Young’s modulus of 376.6 MPa, and an elongation at break of 5.09%. Our work suggests the potential use of highly conductive and mechanically robust ABS-like resin composites in the 3D printing industry. This article not only provides optimized DLP 3D printing conditions for the ABS-like resin, which has both the advantages of the ABS resin and the advantages of a thermoplastic elastomer (TPE), but also presents the effective manufacturing process of ABS-like resin composites with significantly improved conductivity and mechanical properties.

## 1. Introduction

Among various polymer processing methods, 3D printing is becoming a future-oriented polymer processing technology because it can produce customized polymer products of the desired design, shape, and size. 3D-printed polymer products have been used in various applications such as automotive and dental applications, machinery, jewelry, electronics, building materials, and tissue engineering [1,2]. In addition, there are several types of 3D printing methods, such as digital light processing (DLP), stereolithography (SLA), powder bed fusion, material jetting, binder jetting, powder jetting fusion, and material extrusion [1,2]. In particular, DLP 3D printing provides high resolutions in the X–Y plane and *Z*-axis, requires a short printing time, and ensures excellent printing quality and selective light curing [3,4,5,6,7,8,9,10,11,12,13,14,15]. DLP 3D printing does not require the formation of by-products such as supporting materials and enables printing at a distance that needs to be moved hundreds of times when using a laser. In addition, the DLP 3D printer projects a light source to each pixel, enabling accurate lamination on each pixel unit. Therefore, the most significant advantage of DLP 3D printing is that it can quickly produce multiple products with much higher surface quality and accuracy in a shorter time than other 3D printing methods [3,4,5,6,7,8,9,10,11,12,13,14,15]. Moreover, DLP 3D printing supports a variety of polymers, such as lignin [3], epoxy resin [3], acrylate resin (PA) [4,5,6], polyurethane (PU) [7,8,9,10], polycaprolactone (PCL) [10], silicone rubber [11,12], isoprene rubber [13], ABS-like resin [14,15], and so forth. Recently, Chiappone et al. reported the DLP 3D printing of poly(2-hydroxyethyl methacrylate)-*co*-poly(ethylene glycol methyl ether methacrylate) (PHEMA-*co*-PEGMEMA) for use as a shape memory polymer (SMP) stabilized by hydrogen bonding interactions [6]. The urethane acrylate-based resin has been widely studied as a raw material for DLP 3D printing [8,9].

In particular, the ABS-like resin is very suitable for DLP 3D printing because it retains the intrinsic properties of a thermoplastic elastomer (TPE) and provides the unique advantages of ABS, such as processability, adhesion, and mechanical strength. Recently, Liu et al. reported the DLP 3D printing of ABS-like resin composites containing titanium dioxide (TiO_2_) or zinc oxide (ZnO) nanoparticles for use as in antibacterial applications; the ABS-like resin composites employing 1 wt.% of TiO_2_ nanoparticles and 1 wt.% of ZnO nanoparticles demonstrated the maximum tensile strengths of 29.533 and 33.696 MPa, respectively [14]. According to Ji et al., the core part of the omnidirectional scanning mirror for Light Detection and Ranging (LiDAR) applications was fabricated using the DLP 3D printing of ABS-like resin composites [15]. Despite several achievements in the 3D printing of ABS-like resins, there are still limitations, as filler systems that can simultaneously improve the electrical and the mechanical properties of ABS-like resins have yet to be reported. To expand the application field of 3D printable ABS-like resins, optimizing the filler systems so to simultaneously enhance the electrical and the mechanical properties of the ABS-like resins is necessary.

Conducting polymers (CPs) not only retain the inherent properties of polymer materials but also provide unique advantages of tunable electrical conductivity and electrochemical activity, which make them very suitable as fillers that can form conducting networks inside 3D printable resins [16,17,18,19,20]. Among various CPs, polyaniline (PANI) has an excellent redox behavior and allows for reversible doping/dedoping and tunable oxidation levels. Although the production cost of PANI is about one-hundredth of the price of poly(3,4-dioxythiophene) (PEDOT), PANI demonstrates comparable or similar conductivity to PEDOT [18,19,20]. PANI nanomaterials, such as nanofibers [17,18], nanorods [17,18], nanoparticles [17,18], and nanotubes [17,19], offer enlarged surface areas to interact with the polymer interface, which allows them to form denser conductive pathways inside the 3D printable polymer matrix. Accordingly, PANI is considered one of the promising candidates for improving the mechanical and electrical properties of 3D printable resins.

Graphene is a two-dimensional (2D) carbon nanomaterial composed of *sp*^2^-hybridized carbons, which has attractive properties such as high theoretical surface area (2630 m^2^/g), excellent electrical conductivity, high mobility of charge carriers, and high flexibility [20,21,22]. Such interesting features of graphene suggest that it can serve as a filler with very high reinforcing efficiency inside a polymer matrix. Moreover, graphene sheets are widely known to enable additional electron delocalization inside a composite through π−π stacking interactions with PANI nanomaterials [20]. Therefore, the synergistic effect between PANI nanomaterials and graphene-based materials will improve the ABS-like resins’ electrical and mechanical properties. Thus, optimizing the composition of the PANI/graphene co-filler system for the DLP 3D printing of ABS-like resins is necessary.

In this work, PANI nanofibers and graphene flakes were chosen as co-fillers to enhance the mechanical and electrical properties of 3D printable ABS-like resin composites, and the DLP 3D printing technology was applied to achieve improved printing quality and reduced printing time in the 3D printing process of ABS-like resin composite materials. A field emission scanning electron microscope (FE-SEM) was used to investigate the presence and dispersion of PANI nanofibers and graphene flakes within the 3D-printed ABS-like resin composites. Fourier-transform infrared (FT−IR) spectroscopy identified the effects of the PANI/graphene co-fillers on the bonding structures of the 3D-printed composites. The sheet resistance values of the 3D-printed composites showed the reinforcing efficiency of various contents of the PANI/graphene co-fillers in relation to the electrical properties of ABS-like resin composites. Furthermore, the reinforcing effects of the PANI/graphene co-fillers on the mechanical properties of the ABS-like resin composites were studied by determining the stress–strain curves, tensile strength, Young’s modulus, and elongation at break values of the 3D-printed composites.

## 2. Materials and Methods

### 2.1. Materials

Ammonium persulfate (APS, 98%) and aniline (99%) were obtained from Sigma-Aldrich (St. Louis, MO, USA). Water-dispersible graphene flakes were purchased from MExplorer Co., Ltd. (Ansan, Republic of Korea). The graphene flake paste had a density of 25 mg/mL, while the average thickness and lateral size of the graphene sheets were about <5 nm and 2–3 μm, respectively. The ABS-like resin solution (Carima ABS) was purchased from Carima (Seoul, Republic of Korea). Hydrochloric acid (HCl, 35–37%), ethanol (95%), acetone (99%), and isopropyl alcohol (IPA, 99.9%) were purchased from Daejung Chemical & Metals Co., Ltd. (Siheung, Republic of Korea). The PANI nanofibers used in this experiment were synthesized by a modified interfacial polymerization method [17,18,19,20]. A mixture solution was prepared by mixing a 1M aqueous HCl solution (200 mL) with 400 mL of CHCl_3_. Then, 55 mmol of aniline was added to the 1M HCl/CHCl_3_ (1M HCl/CHCl_3_ = 1:2, by volume) mixture, followed by vigorous stirring for 1 h. Then, 26.5 mmol of APS was added to the reaction medium and reacted at 3 °C for 9 h to obtain the PANI precipitates. The as-prepared PANI precipitates were washed with water, ethanol, and acetone.

### 2.2. Fabrication of ABS-like Resin Composites for DLP 3D Printing

Table 1 summarizes the preparative conditions of the ABS-like resin composites. The dispersion of the PANI nanofibers and graphene flakes comprised two steps. In the first step, the resin composite ink containing the PANI nanofibers and graphene flakes was vigorously stirred for 6 h at a stirring speed of 600 rpm. In the next step, the fillers’ dispersion inside the resin composite ink was promoted by sonication for 0.5 h. The sonication treatment of the fillers in the resin composite ink was conducted by using an ultrasonic bath (CPX2800H-E, Branson Ultrasonics Co., Danbury, CT, USA) at 110 W power and 40 kHz frequency. The cold water in the ultrasonic bath was replaced every 0.3 h to maintain the dispersion at room temperature. The viscosity of the as-prepared ABS-like resin composite inks was measured using a rheometer (HR-1, TA Instruments Co., New Castle, DE, USA) at room temperature. The results indicated that the AP5.0 and AP3.0G2.0 samples showed an increase in viscosity of only 8% and 8.8%, respectively, compared to the pristine resin (Table 1). The viscosity results suggested that conducting fillers did not significantly impede the fluid flow inside the ABS-like resins.

### 2.3. DLP 3D Printing and Characterization of ABS-like Resin Composites

The 3D printer used in this work was a DLP-type 3D printing system (IM-96, Carima, Seoul, Republic of Korea), and a 405 nm laser was used as a light source for photo-crosslinking inside the ABS-like resin composites. The ABS-like resin composites containing different amounts of PANI nanofibers and graphene flakes were 3D-printed, and the printability of each sample was evaluated. The maximum printable concentration of PANI was confirmed to be 5.3 wt.% with respect to the resin composite ink, and no lamination occurred when the content of PANI exceeded 5.3 wt.%. The maximum printable concentration of graphene flakes was 2.0 wt.% with respect to the resin composite ink, and no lamination occurred when the content of graphene flakes exceeded 2.0 wt.%. Table 2 summarizes the layer height and exposure time of the ABS-like resin composites obtained through the DLP 3D printing, confirming that the layer height was kept constant at 0.05 mm regardless of the filler composition. A layer height of 0.05 mm was reached in a short exposure time also for the AP5.0 and AP3.0G2.0 samples, in which the filler proportions were increased by 4% and 6%, respectively, compared to the pristine ABS-like resin. This indicated that the presence of conductive fillers did not significantly affect the exposure time required for DLP 3D printing. In order to remove the non-reactive solvent inside the 3D-printed objects, the 3D-printed objects were immersed in IPA and washed for 0.3 h using an ultrasonic bath (CPX2800H-E, Branson Ultrasonics Co., Danbury, CT, USA). Even after this post-treatment, the shrinkage of the 3D-printed sculptures did not occur at all, and the size of the sculptures was maintained even after more than a year. This suggests that appropriate amounts of PANI nanofibers and graphene flakes contribute to maintaining the dimensional stability of 3D-printed sculptures.

A FE-SEM (S-4800, Hitachi, Ltd., Tokyo, Japan) was used to identify the morphologies inside the ABS-like resin composites. A FT−IR spectrometer (Frontier, PerkinElmer Inc., Waltham, MA, USA) was used to investigate the chemical bonds inside the ABS-like resin composites. Ultraviolet–visible (UV–visible) spectra of the ABS-like resin composites were recorded on a UV–visible spectrometer (Mega-U600, Scinco Inc., Seoul, Republic of Korea). The sheet resistance values of the 3D-printed composites were measured using a 4-point probe conductivity meter (Mode Systems Co., Hanam, Republic of Korea) equipped with a current source meter (Keithley 2400, Keithley Co., Cleveland, OH, USA) [16].

**Figure 1:**
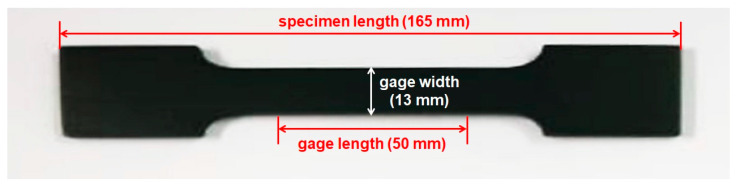
A digital image of a 3D-printed ABS-like resin specimen containing 3 wt.% of PANI and 1.5 wt.% of graphene (AP3.0G1.5).

In order to evaluate the mechanical properties of the 3D-printed composites, tensile tests were performed according to the ASTM standard D638 method using a universal testing machine (UTM, Instron-5543, Instron Co., Norwood, MA, USA) with a crosshead speed of 10 mm/min and a gauge length of 50 mm at room temperature under a relative humidity (RH) of 30% [23]. Each specimen for the tensile tests had a total length of 165 mm, a gauge length of 50 mm, a gauge width of 13 mm, and a thickness of 3.2 mm, and the specimens were easily obtained through DLP 3D printing (Figure 1).

## 3. Results and Discussion

### 3.1. Studies on the Manufacturing and 3D Printing Process of ABS-like Resin Composites

**Figure 2:**
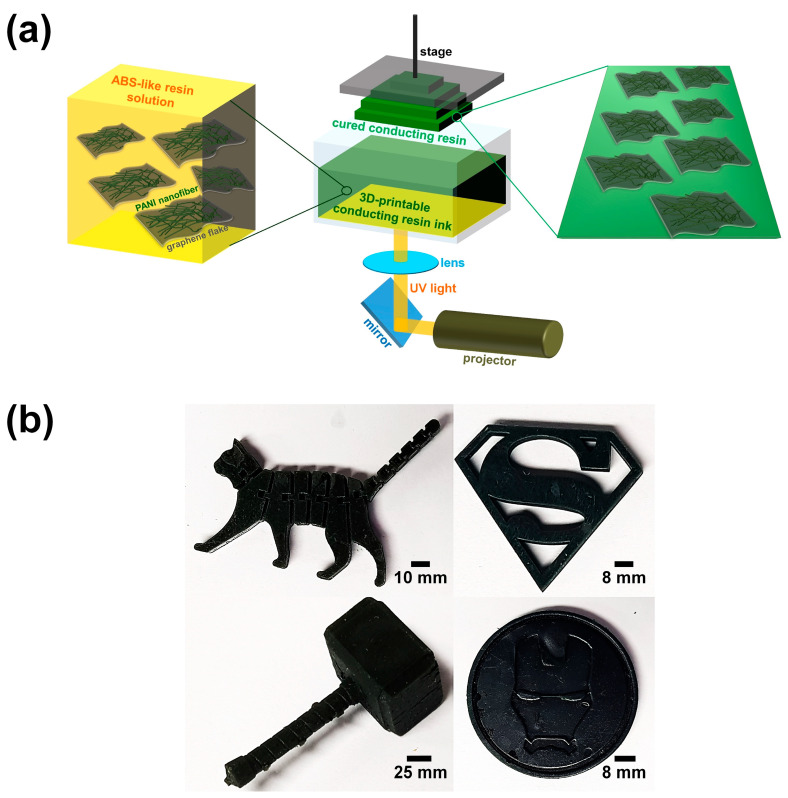
(**a**) Schematic illustration of DLP 3D printing of ABS-like resin composites filled with the PANI/graphene co-filler. (**b**) Digital images of 3D-printed ABS-like resin composites of various shapes and sizes containing 3 wt.% of PANI and 1.5 wt.% of graphene (AP3.0G1.5).

Figure 2a shows a schematic illustration of the DLP 3D printing of ABS-like resin composites. Co-fillers composed of PANI nanofibers and graphene flakes were dispersed in an ABS-like resin solution. These PANI/graphene co-fillers were dispersed in a solution of ABS-like resins containing a cross-linking agent by mechanical stirring and sonochemical treatments. The roles of the PANI nanofibers and graphene flakes in the ABS-like resin were as follows. (1) The PANI nanofibers and graphene flakes, which have high electrical conductivity, formed conjugation paths for electron delocalization inside the insulating ABS-like resin, enabling a high electrical conductivity of the product even after the 3D printing process [17,18,19,20,21,22]; (2) the PANI nanofibers and graphene flakes improved the ultimate mechanical properties of the ABS-like resin after they were evenly embedded inside the ABS-like resin. The ABS-like resin served as a dispersion medium for the PANI/graphene co-fillers and enabled the desired shapes and various sizes of the products obtained after DLP 3D printing [14,15]. The projector present in the DLP-type 3D printer served as a light source supplying UV light (300 nm), which was reflected through a mirror and then passed through a lens to irradiate the ABS-like resin composite ink. After the irradiation of UV light onto the ABS-like resin composite ink, the DLP 3D printing by photo-crosslinking between ABS-like polymer chains proceeded on the stage. Figure 2b shows 3D-printed composites of various shapes and sizes that were actually made. The dispersions of 3.0 wt.% of PANI nanofibers and 1.5 wt.% of graphene flakes were emeraldine and dark gray, respectively, and the final color of the 3D-printed composites was dark green. This indicated that appropriate amounts of PANI nanofibers and graphene flakes contributed to forming ABS-like resin composites with excellent processability without interfering with the resin lamination.

**Figure 3:**
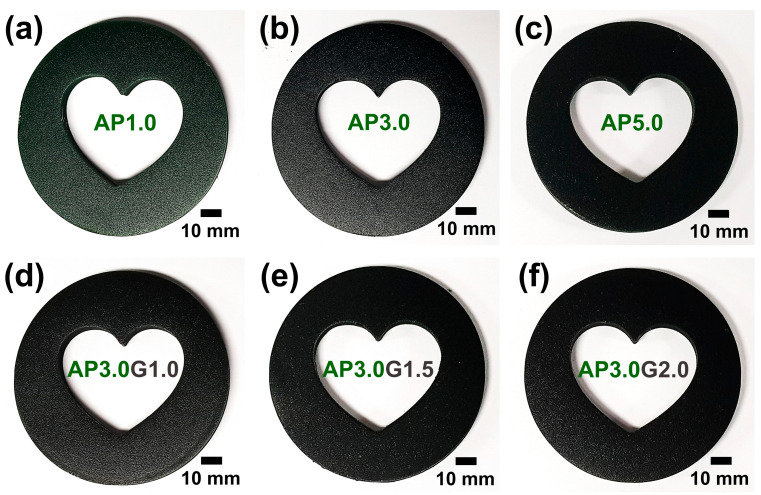
Digital images of 3D-printed ABS-like resin composites with different filler contents: (**a**) AP1.0 (1.0 wt.% of PANI), (**b**) AP3.0 (3.0 wt.% of PANI), (**c**) AP5.0 (5.0 wt.% of PANI), (**d**) AP3.0G1.0 (3.0 wt.% of PANI and 1.0 wt.% of graphene), (**e**) AP3.0G1.5 (3.0 wt.% of PANI and 1.5 wt.% of graphene), and (**f**) AP3.0G2.0 (3.0 wt.% of PANI and 2.0 wt.% of graphene).

Figure 3a–f shows the digital images of the 3D-printed ABS-like resin composites filled with different amounts of PANI nanofibers. It was found that the colors of the 3D-printed composites became darker, indicating the successful loading of PANI nanofibers into the ABS-like resin (Figure 3a–c). In addition, the 3D printing process was successful even when the graphene flakes were added up to 2.0 wt.% to the ABS-like resin composite containing 3.0 wt.% of PANI nanofibers (Figure 3d–f). These results indicated that ABS-like resin composites filled with various amounts of PANI nanofibers and graphene flakes are successfully printable, and the filler contents greatly influence the printability of ABS-like resin composites.

**Figure 4:**
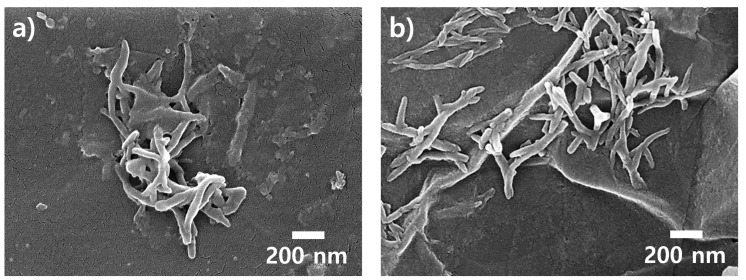
FE-SEM images (magnification: ×50 k) of 3D-printed composites: (**a**) AP3.0 (the composite containing 3.0 wt.% of PANI nanofibers) and (**b**) AP3.0G1.5 (the composite containing 3.0 wt.% of PANI nanofibers and 1.5 wt.% of graphene flakes).

Figure 4 shows FE-SEM images of the AP3.0 (the composite containing 3.0 wt.% of PANI nanofibers) and AP3.0G1.5 (the composite containing 3.0 wt.% of PANI nanofibers and 1.5 wt.% of graphene flakes) samples. In the FE-SEM image of the AP3.0 sample, the diameters and lengths of the PANI nanomaterials were 40−60 nm and 0.6−2.0 μm, respectively (Figure 4a). This proved that the PANI nanofibers were successfully embedded inside the ABS-like resin while preserving the original aspect ratio [17,18]. In dispersing the co-fillers composed of PANI nanofibers and graphene flakes into the resin, the intermaterial PANI−PANI and graphene−graphene attraction forces weakened. In addition, it is known that PANI nanomaterials form π−π interactions with graphene sheets, contributing to additional electron delocalization [20]. Therefore, it was observed that the AP3.0G1.5 sample presented a better dispersibility of the PANI nanofibers inside the resin matrix than the AP3.0 sample (Figure 4b).

### 3.2. Spectroscopic Studies on the 3D Printed ABS-like Resin Composites

**Figure 5:**
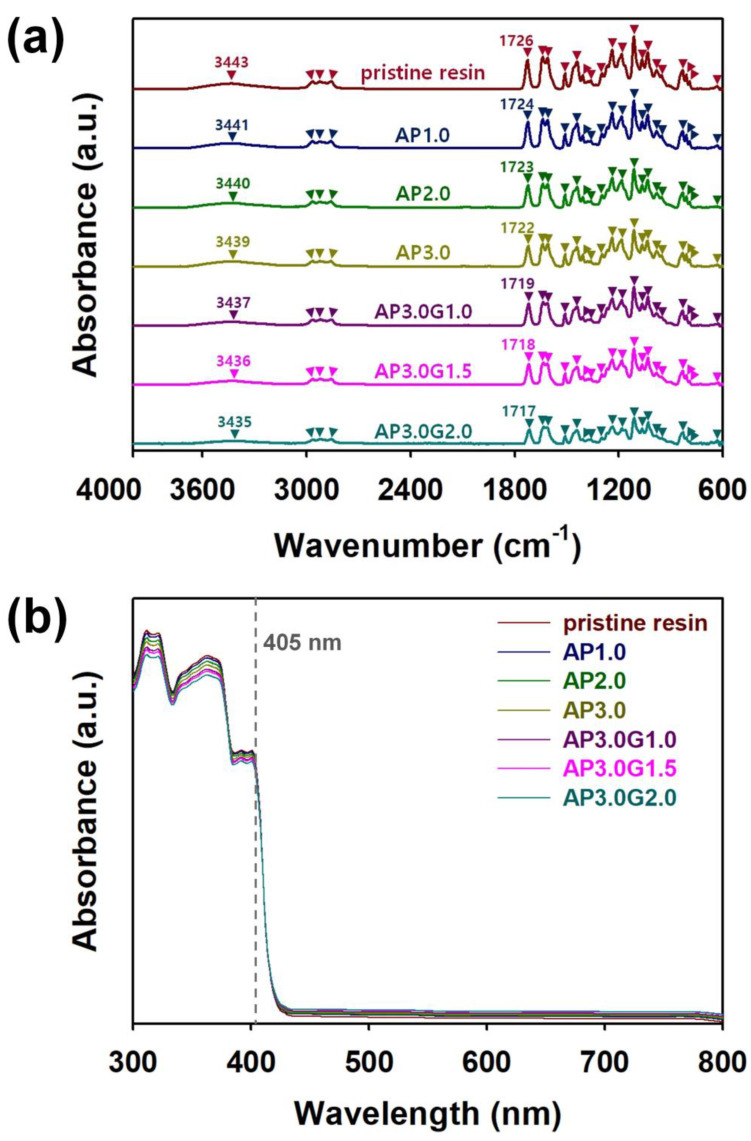
(**a**) FT−IR and (**b**) UV–visible spectra of 3D-printed ABS-like resin composites with different filler contents: pristine resin (dark red line), AP1.0 (dark blue line), AP2.0 (green line), AP3.0 (yellow -green line), AP3.0G1.0 (purple line), AP3.0G1.5 (pink line), and AP3.0G2.0 (turquoise line).

In order to identify the bond structures in the 3D-printed ABS-like resin composites, the FT−IR spectra of ABS-like resin composites filled with different filler amounts were acquired and are shown in Figure 5a. In every FT−IR spectrum of the 3D-printed samples, characteristic peaks for PU were found at the wavenumbers 635, 791, 810, 833, 953, 983, 1032, 1066, 1112, 1183, 1238, 1266, 1297, 1362, 1408, 1441, 1510, 1610, 1639, 1717−1726, 2861, 2927, 2967, and 3435−3443 cm^−1^ (Table S1, see Supplementary Materials) [24,25,26]. This indicated that the main component of the ABS-like resin samples was a PU polymer. Although the characteristic peaks of the PANI nanofibers and graphene flakes were not observed in the FT−IR spectra of the ABS-like resin composites, it was found that the absorbance of the PU peaks was reduced with increasing amounts of the PANI nanofibers and graphene flakes. This indicated the successful incorporation of the PANI nanofibers and graphene flakes into the ABS-like resin matrix. Interestingly, the peaks corresponding to C=O stretching and N-H stretching shifted to lower wavenumbers with increasing amounts of PANI nanofibers and graphene flakes. These blue shifts were found in every FT−IR spectrum of the ABS-like resin composites. These blue shifts suggested that the PANI nanofibers and graphene flakes significantly weakened the attraction forces between ABS-like polymer chains, whereas the attraction forces between ABS-like polymer chains and PANI/graphene co-fillers were further strengthened [26]. In every UV–visible spectrum, the typical absorption peaks of acrylate-based PU were observed at 311, 318, 362, and 385–405 nm (Figure 5b) [27,28]. In contrast, no absorption peaks consistent with graphene flakes and PANI nanofibers were found. This indicated that the fillers were well dispersed inside the ABS-like resin matrix. As the content of PANI nanofibers and graphene flakes increased, the absorption peaks observed at 311, 318, and 362 nm were attenuated. These results are related to the light absorption by the π−π* electron transition of PANI and the n−π* electron transition of graphene in the wavelength range of 300–360 nm [29,30]. In particular, the ABS-like resin composites showed light absorptions at 385–405 nm near the wavelength of the 3D printer’s light source (405 nm), and the attenuation of the absorption peaks at 405 nm was almost infinitesimal even after the filler content increased. This indicated that the PANI nanofibers and graphene flakes did not significantly affect the light-absorbing ability of the ABS-like resin composites during the photo-crosslinking reaction inside the ABS-like resin matrix.

### 3.3. Studies on the Electrical Properties of 3D Printed ABS-Like Resin Composites

**Figure 6:**
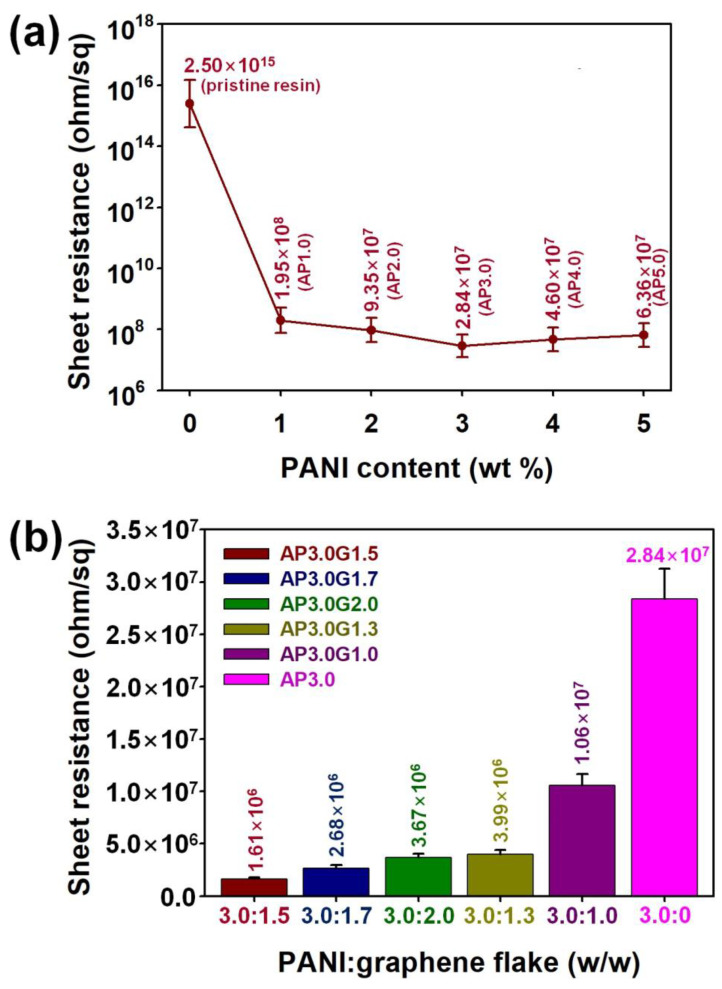
The sheet resistance of 3D-printed ABS-like resin composites with (**a**) different PANI contents and (**b**) different contents of PANI/graphene co-fillers.

Figure 6 summarizes the electrical properties of the 3D-printed ABS-like resin composites filled with different amounts of PANI nanofibers and graphene flakes. The sheet resistance of the 3D-printed composites was reduced from 2.50 × 10^15^ ohm/sq (sheet resistance of the pristine ABS-like resin) to 1.95 × 10^8^, 9.35 × 10^7^, and 2.84 × 10^7^ ohm/sq by adding 1.0, 2.0, and 3.0 wt.% of PANI nanofibers, respectively (Figure 6a). This suggested that the PANI nanofibers formed conducting networks inside the 3D-printed composites, increasing the number of delocalized electrons within the 3D-printed composites [17,18,19,20]. However, the reinforcing efficiency of the PANI nanofibers in relation to the electrical properties of the 3D-printed composites decreased when the PANI content exceeded 3.0 wt.%; in fact, the AP3.0 sample demonstrated an 8.80 × 10^7^ times lower sheet resistance compared to the pristine ABS-like resin, while the AP4.0 and AP5.0 samples exhibited a 5.43 × 10^7^ and 3.93 × 10^7^ times lower sheet resistance, respectively, compared to the pristine ABS-like resin. As the PANI content exceeded 3.0 wt.%, the aggregation of the PANI nanofibers intensified, increasing the intermaterial resistance, which hindered the interfacial interactions between the fillers and the polymer matrix. Based on the above results, the optimal content of PANI nanofibers for maximizing the reinforcing efficiency of the fillers with respect to electrical properties of the 3D-printed composites was set to 3.0 wt.%. Figure 6b represents the sheet resistance of the 3D-printed composites containing 3.0 wt.% of PANI nanofibers and various amounts of graphene flakes as co-fillers. When the co-fillers composed of PANI nanofibers and graphene flakes were introduced into the resin composites, the sheet resistance of the 3D-printed composites became significantly lower than that of the AP3.0 sample. The sheet resistance values of the 3D-printed composites were further reduced from 2.84 × 10^7^ ohm/sq (surface resistance of AP3.0) to 1.06 × 10^7^, 3.99 × 10^6^, and 1.61 × 10^6^ ohm/sq by adding 1.0, 1.3, and 1.5 wt.% of graphene flakes, respectively. When the contents of the graphene flakes were larger than 1.5 wt.%, the reinforcing efficiency of the graphene flakes decreased, as shown for the AP3.0G1.7 and AP3.0G2.0 samples. Accordingly, the AP3.0G1.5 sample demonstrated the lowest surface resistance. This indicated that the synergistic effect of the PANI nanofibers and graphene flakes enabled a better-reinforcing od the electrical properties of the ABS-like resin composites. Considering these results, the appropriate amounts of PANI nanofibers and graphene flakes providing effective conductive pathways within the ABS-like resin composites were confirmed to be 3.0 and 1.5 wt.%, respectively [20,21,22].

### 3.4. Studies on the Mechanical Properties of 3D Printed ABS-Like Resin Composites

**Figure 7:**
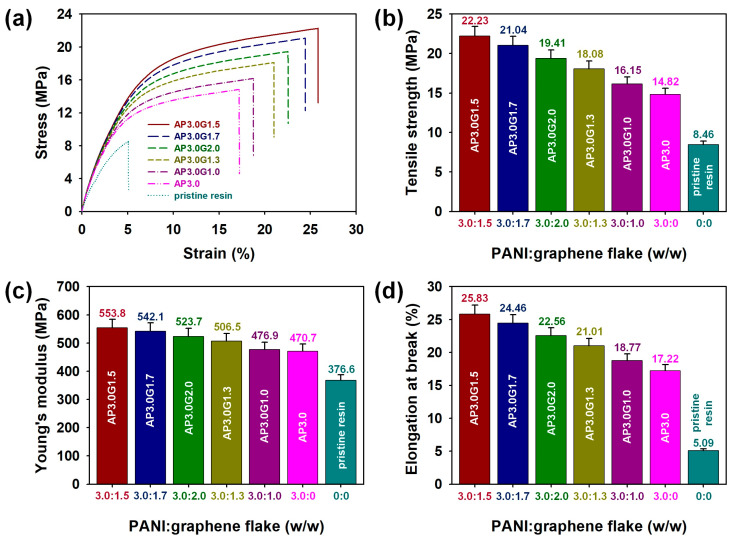
(**a**) Stress–strain curves, (**b**) tensile strength (MPa), (**c**) Young’s modulus (MPa), and (**d**) elongation at break (%) values of the 3D-printed ABS-like resin composites with different contents of PANI/graphene co-fillers. The Young’s modulus (*E*, MPa) was calculated according to the equation *E* = stress (MPa)/strain (%).

To identify the reinforcing effects of the PANI/graphene co-fillers on the mechanical properties of the 3D-printed resin composites, the stress–strain curves of the 3D-printed samples with different filler contents were recorded (Figure 7a). The tensile properties of the samples, such as tensile strength, Young’s modulus, and elongation at break, were determined using a computerized UTM. The tensile strength value of the 3D-printed composites increased from 8.46 MPa (tensile strength of the pristine ABS-like resin) to 14.82 MPa by adding 3.0 wt.% of PANI nanofibers (AP3.0). Furthermore, the tensile strength values of the 3D-printed composites further increased from 14.82 MPa (tensile strength of AP3.0) to 16.15 (8.97% higher compared to that of AP3.0), 18.08 (22.00% higher compared to that of AP3.0), 22.23 MPa (50.00% higher compared to that of AP3.0) by adding 1.0, 1.3, and 1.5 wt.% of graphene flakes, respectively (Figure 7b). The tensile strength of graphene is known to be about 10^5^ to 10^6^ times higher than that of a neat polymer matrix (Table 3) [31]. Therefore, the tensile strength results proved that the reinforcement of the tensile strength inside the ABS-like resin by the graphene sheet was very effective. Thus, the maximum tensile strength was obtained when the content of PANI nanofibers and graphene flakes were 3.0 wt.% and 1.5 wt.% (AP3.0G1.5), respectively. This was likely due to the excellent dispersion of the optimal amounts of PANI nanofibers and graphene flakes and the successful formation of interfacial interactions between the PANI/graphene co-fillers and the ABS-like resin matrix [21,22]. However, when the content of the PANI nanofibers exceeded 1.5 wt.%, the reinforcing efficiency of the graphene flakes in relation to the tensile strength of the ABS-like resins decreased, as shown for AP3.0G1.7 (41.97% higher compared to that of AP3.0) and AP3.0G2.0 (30.97% higher compared to that of AP3.0) samples. This can be probably ascribed to the aggregation of the graphene flakes in the ABS-like resin matrix [21,22]. The Young’s modulus of the 3D-printed composites also demonstrated the maximum value when the contents of PANI nanofibers and graphene flakes were 3.0 wt.% and 1.5 wt.% (AP3.0G1.5), respectively, whereas that of AP3.0G2.0 sample decreased from 553.8 to 523.7 MPa with the increase in the graphene content from 1.5 wt.% to 2.0 wt.% (Figure 7c). Since PANI and graphene led to a Young’s modulus about 10^6^ times higher than that of the neat polymer matrix, the Young’s modulus results obtained from our work can be considered reasonable (Table 3) [31,32]. The elongation at break (%) of the 3D-printed samples was found to increase in the following order: pristine ABS-like resin (5.09) < AP3.0 (17.22) < AP3.0G1.0 (18.77) < AP3.0G1.3 (21.01) < AP3.0G2.0 (22.56) < AP3.0G1.7 (24.46) < AP3.0G1.5 (25.83) (Figure 7d). The significant increase in the toughness of AP3.0G1.5 can be attributed to the excellent dispersion and successful incorporation of the PANI/graphene co-fillers inside the ABS-like resin matrix, as proven by the FT−IR and sheet resistance results (Figure 4 and Figure 5). It is considered that the tensile test results obtained from our work can be cross-validated with “Halpin-Tsai” and “Checkerboard” micromechanics models in future work [33,34,35,36].

## 4. Conclusions

In this study, we successfully developed the DLP 3D printing of ABS-like resin composites containing co-fillers composed of PANI nanofibers and graphene flakes. The ABS-like resin composites containing the PANI/graphene co-fillers could be 3D-printed as conducting sculptures with different sizes and desirable shapes. More importantly, we optimized the composition of the PANI/graphene co-filler to maximize the electrical and mechanical properties of the ABS-like resin composites. Significant enhancement of the electrical properties could be achieved in the AP3.0G1.5 sample (the composite containing 3.0 wt.% of PANI nanofibers and 1.5 wt.% of graphene flakes) compared to the pristine ABS-like resin. Furthermore, the mechanical properties of the 3D-printed composites were found to be significantly improved by adding 3.0 wt.% of PANI nanofibers and 1.5 wt.% of graphene flakes into the ABS-like resin matrix. The enhanced electrical and mechanical properties could be attributed to the successful formation of a conductive network and the improved dispersion of the filler within the ABS-like resin matrix due to the synergistic effect of the PANI nanofibers and graphene flakes. The novelty of our study is that it provides manufacturing conditions and successful 3D printing conditions for composites that can improve the conductivity and mechanical properties of ABS-like resins, a TPE material with the advantages of the ABS resin. Although specific applications of the ABS-like resin composites were not presented in this article, such highly conductive and mechanically robust ABS-like resin composites could be widely used as essential materials for electronic devices, automotive parts, thermal pads, thermoelectric devices, sensors, and energy storage devices in the future.

## Figures and Tables

**Table 1:** Preparative conditions of ABS-like resin composite inks.

Sample	ABS-like Resin (g)	PANI Nanofiber (g)	Graphene Flake (g)	Viscosity (cps)
pristine	20.00	-	-	375 ± 25
AP1.0	19.80	0.20	-	385 ± 30
AP2.0	19.60	0.40	-	390 ± 30
AP3.0	19.40	0.60	-	395 ± 30
AP4.0	19.20	0.80	-	400 ± 35
AP5.0	19.00	1.00	-	405 ± 35
AP3.0G1.0	19.20	0.60	0.20	398 ± 30
AP3.0G1.3	19.14	0.60	0.26	401 ± 35
AP3.0G1.5	19.10	0.60	0.30	403 ± 35
AP3.0G1.7	19.06	0.60	0.34	405 ± 35
AP3.0G2.0	19.00	0.60	0.40	408 ± 35

**Table 2:** Three-dimensional printing conditions for ABS-like resin composite inks.

Sample	Layer Height (mm)	Exposure Time (s)
pristine	0.05	5.0
AP1.0	0.05	5.1
AP2.0	0.05	5.1
AP3.0	0.05	5.1
AP4.0	0.05	5.2
AP5.0	0.05	5.2
AP3.0G1.0	0.05	5.2
AP3.0G1.3	0.05	5.2
AP3.0G1.5	0.05	5.2
AP3.0G1.7	0.05	5.3
AP3.0G2.0	0.05	5.3

**Table 3:** Tensile properties of each component.

Sample	Tensile Strength (MPa)	Elongation at Break (%)	Young’s Modulus (MPa)
ABS-like resin	8.46	5.09	376.6
graphene	1.30 × 10^6^ [31]	-	1.00 × 10^9^ [31]
polyaniline	-	-	9.00 × 10^8^ [32]

## Data Availability

Not applicable.

## Supplementary Materials

The following supporting information can be downloaded at: https://www.mdpi.com/article/10.3390/polym15143079/s1, Table S1: Characteristic bands with specific vibrational modes of the ABS-like resin composites.
