# Supplementary material for: The Effects of Polyaniline Nanofibers and Graphene Flakes on the Electrical Properties and Mechanical Properties of ABS-like Resin Composites Obtained by DLP 3D Printing
Source: Polymers (Basel). 2023 Jul 18;15(14):3079. doi: 10.3390/polym15143079 (PMC10386287; doi:10.3390/polym15143079)
Supplement: Supplementary file 1 [file polymers-15-03079-s001.zip › polymers-2280109-supplementary.pdf]

Supplementary

The Effects of Polyaniline Nanofibers and Graphene Flakes on the Electrical Properties and Mechanical Properties of ABS-like Resin Composites Obtained by DLP 3D Printing

Somi Jang [1,2] and Sunghun Cho [1,*]

[1] School of Chemical Engineering, Yeungnam University, Gyeongsan 38541, Republic of Korea
[2] Department of Materials Science and Engineering, Research Institute of Advanced Materials, Seoul National University, Seoul 08826, Republic of Korea
* Correspondence: shcho83@yu.ac.kr; Tel.: +82-53-810-2535

Table S1. Characteristic bands with specific vibrational modes of ABS-like resin composites.

Wavenumber (cm^{-1})	Vibration
635, 791, 810	C=C out-of-plane bending
833, 953, 983	C–H out-of-plane bending
1032, 1066	C–O–C symmetric stretching
1112	C–O–C asymmetric stretching
1183	C–H in-plane bending, C–C stretching, C–O–C asymmetric stretching
1238, 1266, 1297, 1362, 1408, 1441, 1510	C–N stretching, C=N in-plane bending, C–H in-plane bending, N–H bending
1610, 1639	C=C stretching
1717–1726	C=O stretching
2861	C–H symmetric stretching
2927, 2967	C–H asymmetric stretching
3435–3443	N–H stretching
